# Haematogenous *Staphylococcus aureus *meningitis. A 10-year nationwide study of 96 consecutive cases

**DOI:** 10.1186/1471-2334-6-49

**Published:** 2006-03-16

**Authors:** Michael Pedersen, Thomas L Benfield, Peter Skinhoej, Allan G Jensen

**Affiliations:** 1 Department of Infectious Diseases, Copenhagen University Hospital, Rigshospitalet, Blegdamsvej 9, 2100 Copenhagen, Denmark; 2 Staphylococcal Laboratory, Statens Serum Institut, Artillerivej 5, 2300 Copenhagen, Denmark; 3 Department of Clinical Microbiology, Copenhagen University Hospital, Rigshospitalet, Blegdamsvej 9, 2100 Copenhagen, Denmark

## Abstract

**Background:**

Haematogenous *Staphylococcus aureus *meningitis is rare but associated with high mortality. Knowledge about the disease is still limited. The objective of this study was to evaluate demographic and clinical prognostic features of bacteraemic *S. aureus *meningitis.

**Methods:**

Nationwide surveillance in Denmark from 1991 to 2000 with clinical and bacteriological data. Risks of death were estimated by Cox proportional hazards regression analysis.

**Results:**

Among 12480 cases of *S. aureus *bacteraemia/sepsis, we identified 96 cases of non-surgical bacteraemic *S. aureus *meningitis (0.8%). Incidence rates were 0.24 (95% confidence interval [CI], 0.18 to 0.30)/100 000 population between 1991–1995 and 0.13 (CI, 0.08 to 0.17)/100 000 population between 1996–2000. Mortality was 56%. After adjustment, only co morbidity (hazard ratio [HR], 3.45; CI, 1.15 to 10.30) and critical illness (Pitt score ≥ 4) (HR, 2.14; CI, 1.09 to 4.19) remained independent predictors of mortality.

**Conclusion:**

The incidence, but not mortality of bacteraemic *S. aureus *meningitis decreased during the study period. Co morbidity and critical illness were independent predictors of a poor outcome.

## Background

Spontaneous, non-surgical meningitis caused by *Staphylococcus aureus *is a rare disease with an annual incidence in Denmark of 0.22 per 100 000 population[1]. Mortality rates (50%) are higher than for other types of bacterial meningitis [1-3]. Age, diabetes mellitus, disseminated intravascular coagulation[4], impaired consciousness on presentation[4,5], presence of shock and infection with phage type 95[1] have all been associated with a poor outcome by an univariate statistical analysis.

Two different modes of *S aureus *meningitis pathogenesis have been considered in the literature: hospital-acquired infection with a positive blood culture and evidence of infection later than 48 hours after admission, often associated with either head trauma or neurosurgical procedures, or a community-acquired infection with a positive blood culture and evidence of infection less than 48 hours after admission, in the absence of any recent head trauma or surgical procedures i.e. due to an infection outside the central nervous system[1,3-6]. Most studies have evaluated both surgically introduced and spontaneous *S aureus *meningitis cases[1,3-6]. However, there is a considerable difference between the two groups, e.g. as regards mortality, age and frequency of metastatic infections[1,3,4,7]. To our knowledge there has never been a review of a large number of haematogenous i.e. community-acquired bacteraemic *S aureus *meningitis cases exclusively.

Predictors of meningitis and outcome have an impact on patient management. The proper antibiotic treatment has been considered in several studies[1,3-5,8]; however, the optimal antibiotic recommendation remains unclear. Here we evaluate demographic features and clinical variables of prognostic influence on the outcome of haematogenous *S aureus *meningitis.

## Methods

### Selection of cases

The Staphylococcal Laboratory, Statens Serum Institut, Copenhagen, annually receives almost all *S aureus *blood culture strains for phage typing and resistance surveillance from all clinical microbiology departments in Denmark. In addition discharge summaries from all episodes are collected. In cases with clinical evidence of meningitis and verified *S aureus *aetiology, the medical records were obtained from the relevant clinical department and reviewed. The diagnosis was based on the following criteria: (i) characteristic clinical findings; (ii) bacterial cultures of CSF and/or blood showing *S aureus*; (iii) CSF leukocyte count > 50 * 10^6^/l with > 80% neutrophils. In cases with a negative CSF culture, a positive blood culture with *S aureus *and CSF pleocytosis was considered indicative for *S aureus *meningitis [5]. Cases with clinical findings were included if an identical strain was identified in CSF and blood cultures. Population data was obtained from Danmarks Statistik [9].

### Definitions and clinical data

For all cases the following data were registered: Age, sex, blood cultures results, overall survival or in-hospital death of the patient, old people's home residence (a home outside of the hospital for people over 65 years of age who require full-time care). Alcohol consumption, tobacco use, symptoms and clinical signs, temperature, blood pressure, heart rate, which was uniformly taken from the time of admittance to the hospital. Recent surgery (< 4 weeks), primary and secondary focus. Primary focus of infection was based on evident clinical signs and/or symptoms that were later confirmed by cultivation of a bacterial strain with the same resistance pattern as the blood culture strain. Endocarditis, osteomyelitis, arthritis and meningitis were considered secondary foci if the patient had not received surgical intervention at these sites prior to the onset of *S aureus *bacteremia. Co morbidity was divided in categories of competing illnesses. They were cardiac illness, pulmonary illness, diabetes, alcoholism, neurological illness, reumatological illness, renal illness, gastrointestinal malignancy and other immunosuppression. Co morbidity was registered and for the statistical calculations divided in none, one or more than one predisposing condition.

### Classification of meningitis cases and exclusions

Meningitis was classified as community-acquired, nosocomial or health care associated. Meningitis was defined as community acquired, if diagnosed with a positive blood culture < 48 hr. after hospitalisation in patients with no regular contact with the hospital, with clinical evidence of infection and if the patient had not received surgical intervention within the preceding four weeks. Nosocomial meningitis was defined as a hospital acquired meningitis > 48 hr. after hospitalisation. Meningitis was defined as health care associated, if diagnosed < 48 hr. after hospitalisation in patients with regular contact with the hospital e.g. in an out-patients' clinic. It the cases were not classifiable in one of the three above-mentioned categories they were excluded from the study.

### Laboratory data

Also a registration of date of the lumbar puncture, cerebrospinal fluid (CSF) microscopy and culture, CSF glucose, CSF protein, serum glucose, CSF cell count and the blood biochemistry variables pH, red and white blood cell count, platelets, CRP, potassium, sodium, creatinine, and carbamide was done. All the different image diagnostics were listed.

### Severity of illness

Whether there was a need for transfer to intensive care unit, for vasopressor support, mechanical ventilation or dialysis was noted. An APACHE (Acute Physiology, Age and Chronic Health Evaluation) II score could not be performed retrospectively. Instead we calculated the Pitt bacteraemia score that assesses the severity of illness[10].

### Antimicrobial treatment

Initial antibiotic treatment and duration, whether the initial treatment was relevant and eventual change of antibiotics was registered together with bacterial resistance. Initial treatment was defined as the first antibiotic therapy the patient received when submitted to the hospital i.e. prior to the microbiological verification of the bacterial infection. The treatment was considered appropriate if the *S aureus *strain was found susceptible to the antimicrobial treatment administered.

### Bacteriology

Susceptibility to antibiotics of the infecting strains was determined by a disk diffusion method[1]. The testing comprised susceptibility to penicillin, streptomycin, tetracycline, erythromycin, methicillin, fusidic acid, ciprofloxacine and gentamicin.

### Statistical analysis

All values are expressed as median and range. Differences between groups were estimated by Mann-Whitney test or χ^2 ^statistics, as appropriate. P < 0.05 was considered significant. Cox proportional hazard regression analysis was used to estimate hazard ratio associated with outcome. The likelihood ratio test was used to test for interaction. There were no significant interactions. The adequacy of the model was checked by testing the proportional hazards assumption in different ways: by conducting the traditional graphics check based on the log of the cumulative hazard and by performing a formal test of proportionality based on Schoenfeld residuals according to the method of Hosmer and Lemeshow [11]. Statistical analysis was performed with SPSS 11.5 (Statistical Package for Social Sciences, Chicago, IL.) and Stata Statistical Software (version 9.0; Stata Corporation, College Station, TX). P < 0.05 on a two-sided test was considered significant.

### Limitations of study design

Cases were identified prospectively but detailed chart data was extracted retrospectively. Therefore, some factors that influence outcome may not have been captured and we cannot exclude the possibility of residual confounding. Although, this is the largest published data set of S aureus meningitis, the low number of cases may have limited the multivariate analysis.

### Ethical approval

The study was approved by the ethical committee for Copenhagen and Frederiksberg Counties (01-369/93).

## Results

### Patient characteristics

During the whole study period from 1991 to 2000, 12480 cases of *S. aureus *bacteraemia were received. Of these, 107 patients with bacteraemic *S. aureus *meningitis were identified. Eleven cases were surgical infections and therefore excluded from analysis. Of the remaining 96 patients with haematogenous *S. aureus *meningitis, 81 cases were community-acquired, ten cases were nosocomial and five cases health care associated. The total incidence for the period 1991–2000 was 0,18 (95% confidence interval [CI], 0,15–0,22)/100 000 population. However, the incidence declined through the study period. For the period 1991–1995 it was 0,24 (CI, 0,18–0,3)/100 000 population and for 1996–2000 it was 0,13 (CI, 0,08–0,17)/100 000. The sex distribution was 42 (44%) men and 54 (56%) women. Although the median age was high (67 years, range (0–97)), only two patients came from an old people's home and 12 patients were intravenous drug users. In 95 assessable cases, 58 (61%) had an unknown primary focus, whereas 39% had foci in skin, lungs or urinary tract. In 57 cases there were a secondary focus in addition to meningitis and of these patients, 35 (61%) had endocarditis, 17 (30%) had osteomyelitis and 5 (9%) had other secondary focus. Of 64 patients, 34 (53%) were smokers and 13 of 61 patients (21%) consumed excess amounts of alcohol. Of 95 patients 11 (12%) had diabetes. At presentation the majority of the patients (85%; 71/84 patients) had fever, 24% (22/92) had headache, 17% (16/92) had vomiting and 4% (4/92) had seizures. Of 89 patients, 66 (74%) had changes in mental status, 58 (65%) had nuchal rigidity and 17 (19%) focal neurological findings. 20% (18/89) had a rash. 56% (54/96) of the patients died during admittance.

### Laboratory characteristics at admission

Laboratory characteristics of the patients are shown in Table 1. Temperature and paraclinical findings were not significantly different between survivors and non-survivors, except for blood-glucose, which was lower for survivors (median, 6,9 (range, 5–11) mmol/l vs. median 8,4 (range, 6–35) mmol/l) (N = 36) (P = 0,048) (Table 1). The cerebrospinal fluid white blood cell count was 148 * 10^6^/l (range, 1–13870 * 10^6^/l) (N = 66), with 90% polymorph nuclear neutrophils (range, 40–100%) (N = 48). In 4 cases the lumbar puncture results were unavailable. *S aureus *was seen in the spinal fluid in 15 of 55 (27%) cases by microscopy. Of 73 cases with available information, 53 (73%) had a spinal fluid with a positive bacterial culture.

**Table 1:** Demographic and Laboratory Characteristics of Patients with *S. aureus *meningitis according to survival.

Chraracteristics	All Patients (N = 96)	Survivors (N = 42)	Non-survivors (N = 54)	P value
Age, yr	67 (0–97)	54 (0–97)	72 (35–90)	<0.001
Male/female gender, No	42/54	20/21	22/33	= 0.50
Cell count, total	149 (1–13870)	105 (2–2410)	196 (1–13870)	= 0.097
Cell count, %polymorphnuclear	90 (40–100) (N = 46)	86 (40–100) (N = 19)	90 (50–100) (N = 27)	= 0.47
S-glucose, mmol/l	7.6 (5–35) (N = 36)	6.9 (5–11) (N = 15)	8.4 (6–35) (N = 21)	= 0.048
CRP, mg/l	245 (25–459) (N = 32)	258 (25–392) (N = 15)	222 (138–459) (N = 17)	= 0.48
Initial treatment, No				
Relevant	34	16	18	
Irrelevant	53	20	33	= 0.27
Pitt score≥4, No	56	16	40	= 0.006
Other secundary focus, No	57	28	29	= 0.35
Intra-veneous drug use, No	12	9	3	= 0.028
Comorbidity, No				
None	26	5	21	
1	36	24	12	
>1	35	26	9	<0.001

Unless otherwise indicated, data are presented as No. or median (range).

### Treatment of meningitis

As expected in Scandinavia, all but one of the 96 *S aureus *strains were methicillin-sensitive (MSSA), whereas 87 (90%) were resistant to penicillin, three strains were resistant to tetracycline, two to ciprofloxacin, two to streptomycin and one to erythromycin. The initial empiric treatment is illustrated in Table 2. Initially, 57 (61%) of the 93 patients were treated with penicillin or ampicillin with or without an amino-glycoside. In 3 cases information about the initial treatment were unavailable. The initial treatment was considered inappropriate in 53 (61%) of the 87 cases, since all 53 strains were penicillin resistant and aminoglycoside was not expected to sterilize CSF. Of these, 33 (62%) died. The initial treatment was appropriate in 34 (39%) of the cases and among these, 18 (53%) died. In 9 cases it was not possible to evaluate whether the initial treatment was relevant. However, there was no significant difference in survival between patients who received an appropriate and an inappropriate treatment (p > 0,2) (N = 87). When the diagnosis of *S aureus *meningitis was established, the treatment was changed in 81 (84%) of the 96 cases to specific anti-staphylococcal therapy. After bacterial diagnosis 41 (77%) of the initial 53 patients receiving an inappropriate treatment were change to an appropriate treatment. After bacterial diagnosis and susceptibility testing, 82 (90%) of the 91 patients then received an appropriate antibiotic treatment and 9 (10%) an inappropriate treatment. There was no significant difference in mortality between the two groups (p > 0,5) (N = 91). In 5 cases there were no information about the antibiotic treatment after the bacterial diagnosis.

**Table 2:** Initial antibiotic treatment in S. aureus meningitis.

Antibiotics	Number, total	Number of deaths
**Inappropriate antibiotic treatment:**		
Ampicillin	4	2
Ampicillin+gentamicin	12	5
Erythromycin	1	1
Penicillin	32	22
Penicillin+gentamicin	3	2
Ampicillin+cefotaxim*	1	1
Mortality in the group		62%

**Relevant antibiotic treatment:**		
Methicilline	3	1
Methicilline+fusidic acid	2	1
Dicloxacillin	4	0
Dicloxacillin+rifampin	1	1
Dicloxacillin+fusidic acid	1	0
Ceftriaxone	12	5
Vancomycin+ceftriaxone	1	1
Vancomycin+fusidic acid	1	1
Cefuroxime	3	3
Ampicillin**	3	3
Ampicillin+chloramphenicol**	1	0
Penicillin**	2	2
Mortality in the group		5%

Total, No.	87	51

* MRSA strain. ** Penicillin sensitive strains. Mortality between the two groups is not significantly different (P > 0,2).

### Mortality

The importance of risk factors for mortality in univariate and multivariate analysis are shown in Table 3. Fifty-four of the 96 patients died (56%). To explore the risk of progression to death associated with baseline variables, each variable was entered in a Cox regression model by univariate analysis. Age above 65 yr., co-morbidity (one or more predisposing diseases) and critical illness defined by Pitt score >3 each were univariately associated with an increased mortality, while intravenous drug use was univariately associated with a decreased mortality (Table 3). The following variables were not associated with mortality: alcohol consumption, tobacco use, portal of entry of the bacteria, vasoactive support, mechanical ventilation, dialysis, and whether the patient was recently operated. Also, there was no relation between mortality and endocarditis or osteomyelitis compared with the group not having endocarditis or osteomyelitis. By multivariate analysis only co morbidity and critical illness remained independent predictors of survival (Table 3).

**Table 3:** Uni- and multivariate analysis of risk factors associated with in-hospital death from *S aureus *meningitis.

	Univariate HR (95% CI)	Multivariate HR (95% CI)	p value (Multivariate)
**Age, > 65 years**	2.65 (1.47–4.78)	1.57 (0.79–3.13)	0.20
**Comorbidity**	5.13 (2.03–12.96)	3.45 (1.15–10.30)	0.03
**Intravenous drug use**	0.31 (0.10–0.99)	0.64 (0.16–2.53)	0.53
**Critical illness **(Pitt score > 3)	2.58 (1.37–4.93)	2.14 (1.09–4.19)	0.03

HR: hazard ratio. CI: confidence interval. Cox regression with all variables foced into the model. HRs are adjusted with all variables including in the model.

## Discussion

As the incidence of both community-acquired and nosocomial *S aureus *bacteraemia increased during the study period, we anticipated an increase in haematogenous *S aureus *meningitis cases, but observed a reduction. This could be explained by either an earlier detection and treatment of *S aureus *bacteraemia thus preventing spread, misdiagnoses or an unbalanced under-reporting of the meningitis cases[12]. Of note, this series only comprise bacteraemic cases, which is estimated to be only 80% of all *S aureus *meningitis cases[1]. *S aureus *meningitis is an uncommon infection, accounting for approximately 2,6% of all bacterial meningitis[5], but the number of cases is probably underreported[12].

During a ten-year period from 1991 to 2000, we identified 96 cases of spontaneous *S aureus *meningitis, which to our knowledge is the largest reported series of haematogenous *S aureus *meningitis. These cases occurred among 12480 patients with *S aureus *bacteraemia (0,8%). As also reported by other groups, the patients have an advanced age and a short history of symptoms before admission to hospital[1,5]. Contrary to other *S aureus *meningitis studies, using univariate analysis, we do not find age to be a risk factor for death[1,4,5,7,8]. Also in coagulase-negative staphylococcal meningitis[13], tuberculosis meningitis[14] and *Streptococcus pneumoniae *meningitis[15] advanced age has been found to be associated with poor outcome by univariate analysis. However, patients with advanced age have more competing illnesses[16], which could explain that age is not significant in a multivariate test, as competing illnesses might have a larger impact on the patients overall health status when having meningitis.

*S aureus *may only penetrate/cross the blood brain barrier with difficulty. Studies suggest that *S aureus *is a non-invasive extracellular pathogen that damages host cells after adhering to the extra cellular matrix[17]. However, infections with large bacterial loads and of considerable duration may promote development of meningitis. In support of this, a significant number of the patients (60%) had other secondary focuses such as endocarditis (36%) or osteomyelitis (16%), making these conditions important risk factors for meningitis. Patients with either *S aureus *endocarditis or osteomyelitis characteristically present non-specific, vague and diffuse symptoms[18,19]. Therefore, the duration of time the microorganisms (*S aureus*) are present in the blood could be of importance, as has been found for tuberculosis meningitis[14], even if the bacterial count is low[20]. Also, an earlier study shows that patients with endocarditis have an increased mortality if they have neurological manifestations, including meningitis[21]. This supports a recommendation that patients with *S aureus *meningitis should all be examined for both endocarditis[21,22] and osteomyelitis[23]. In our patients, however, simultaneous endocaditis or osteomyelitis did not influence survival.

Diabetes mellitus is associated with increased rates of *S aureus *colonization and infection risk[18]. In the present study survivors had lower blood glucose than the non-survivor, but this could not be estimated in multivariate models because only a minority had available blood glucose values. In our series there were more patients with hyperglycaemia than had a known diagnosis of diabetes (Table 1). Whether the patients had hyperglycaemia as a predisposing factor to the infection or whether they have hyperglycaemia as a consequence of the stress response caused by the massive infection, the data did not indicate further[24]. Diabetes mellitus is a predictor of poor outcome in earlier studies of both *S aureus *bacteraemia and meningitis [25-27]. Hyperglycaemia is known to be associated with increased mortality and significant impairment in functional recovery in patients with cerebral ischaemia[28]. The mechanism is not well defined, but hyperglycaemia does induce cerebral oedema and blood-brain-barrier defect[29]. It is possible that a tight glycaemic control in these patients may be beneficial and should be the subject of future research[29].

The appropriate antibiotic treatment for *S aureus *meningitis has been debated through out the literature[1,3,4,8,12,30]. In spite of the many cases, our case series proved to be to small for a recommendation by multivariate analysis. Initially the majority of the patients received an inappropriate antibiotic anti-staphylococcal treatment due to the fact that staphylococcal infection was not suspected and that, in Denmark, the guidelines recommend a penicillin for empirical treatment of infections prior to susceptibility testing, because a community-acquired infection usually predicts susceptibility penicillins [12]. Therefore, as for outcome, the optimal anti-staphylococcal treatment remains unclear.

Mortality is still very high (56%) and unchanged compared to earlier *S aureus *meningitis surveys[1,3,19]. In large surveys of adults with bacterial meningitis, pneumococcal meningitis is emphasized because of mortality rate of approximately 30 percent[2,31]. Although *S aureus *meningitis cases are few in numbers[1,12,31], they should be considered as a possible aetiology in the initial management in order to improve outcome[32]. Knowledge of the causative organism of meningitis is important in predicting the risk of an unfavourable outcome. Patients are critically ill at admission, but there was no direct association between survival and admission to an intensive care unit in this study. Co morbidity and critical illness (Pitt score ≥ 4) were independently associated with risk of death. The prognosis was poor both if the patient had just one predisposing condition and if there where more than one. It is of importance for outcome whether the patients are seriously chronic ill at the onset of meningitis, a finding that Mylotte and Tayara[26] find in a cohort of *S aureus *bacteraemia. Special attention should be given patients with the presence of underlying disease, as this risk factor is strong in our study and in other studies of both bacterial meningitis in general and of *S aureus *meningitis[7,31].

## Conclusion

The incidence of *S aureus *meningitis decreased during the study period, although the number of bacteraemia increased. By multivariate analysis only co morbidity and critical illness were independent predictors of outcome. Advanced age was not a risk factor for death. Our recommendations to improve diagnosis and potentially the prognosis of patients with *S aureus *meningitis would be, to evaluate patients who have a positive *S aureus *culture or with a suspicion of invasive *S aureus *infection for signs and symptoms of meningitis. Verified cases should be closely evaluated for other secondary foci especially endocarditis and osteomyelitis.

## Competing interests

The author(s) declare that they have no competing interests.

## Authors' contributions

MP drafted the manuscript, participated in the study design and the statistical analysis. TLB performed the statistical analysis and helped to draft the manuscript. PS participated in the coordination of the data collection and help draft the manuscript. AGJ participated in the design and coordination of the data collection. All authors read and approved the final manuscript.

## Pre-publication history

The pre-publication history for this paper can be accessed here:


